# Skeletal Class III Camouflage Using Carriere^®^ Motion 3D and Clear Aligners: A Hybrid Case Report Approach

**DOI:** 10.3390/reports9010046

**Published:** 2026-01-31

**Authors:** Luis Huanca Ghislanzoni, Claudia Lapprand, Thomas Mourgues

**Affiliations:** 1 Division of Orthodontics, University Clinics of Dental Medicine, University of Geneva, 1211 Geneva, Switzerland; 2 Department of Orthodontics, Alfonso X University, Villanueva de la Cañada, 28691 Madrid, Spain; 3 Department of Orthodontics, Rey Juan Carlos University, 28922 Alcorcón, Spain

**Keywords:** class III malocclusion, orthodontic camouflage, carriere motion appliance, clear aligners, case report

## Abstract

**Background and Clinical Significance**: Treating severe skeletal Class III malocclusions in adults who refuse orthognathic surgery remains challenging. Orthodontic camouflage offers a non-surgical option to improve occlusion and esthetics. **Case Presentation**: A 26-year-old male with a full bilateral Class III malocclusion and anterior crossbite was treated following the “Sagittal First” philosophy. The Carriere^®^ Motion 3D Class III appliance was used for mandibular distalization, combined with active maxillary aligners and Class III elastics. After 32 months, a stable Class I occlusion with proper overjet, overbite, and improved sagittal balance was obtained. Cephalometric analysis showed clockwise mandibular rotation and satisfactory dentoalveolar compensation. **Conclusions:** Combining the Carriere^®^ Motion 3D appliance with clear aligners can successfully camouflage severe skeletal Class III malocclusions in adults, providing a predictable and esthetic non-surgical alternative.

## 1. Introduction and Clinical Significance

Class III malocclusions display considerable clinical and etiological variability, often involving a combination of skeletal, dentoalveolar, and functional imbalances [1,2]. In adult patients, once growth is complete, the treatment of severe cases typically relies on a surgical approach to reestablish maxillomandibular relationships [3,4]. However, an increasing number of patients decline surgery due to postoperative constraints, cost, or personal considerations, prompting clinicians to adopt a purely orthodontic compensatory approach [5].

Orthodontic compensation of skeletal Class III cases aims to camouflage the skeletal discrepancy by retroclining the mandibular incisors, proclining the maxillary incisors, and correcting transverse and vertical imbalances [6,7]. This strategy requires precise anchorage control and a thorough assessment of the biological limits of root and alveolar movement, particularly to avoid periodontal side effects such as gingival recession or bone dehiscence [8]. As this type of treatment offers limited improvement to the facial profile, the objectives are generally confined to enhancing functional occlusion, dental esthetics, and masticatory comfort.

The development of systems combining skeletal anchorage, distalization devices, and clear aligners has broadened therapeutic possibilities, even in cases traditionally considered surgical. The Carriere^®^ Motion Class III appliance, used in the initial “sagittal first” phase in conjunction with passive clear aligners in the opposing arch, provides a useful tool for early correction of anteroposterior discrepancies through controlled mandibular distalization, associated with a later treatment with fixed appliances [9,10,11]. In this context, more recently, Lombardo et al. suggested the use of active aligners both as anchorage and as an active tool not only for the finishing phase, but also for the initial anchorage phase, offering the possibility of earlier control of tooth movements, with particular attention to incisor torque [12].

This clinical case illustrates the implementation of such a strategy in the non-surgical management of complete skeletal Class III malocclusion in an adult patient. The approach involved an initial sagittal correction with the Carriere^®^ Motion appliance, active clear aligners, and a partially hybridized treatment plan to address specific anatomical and mechanical constraints.

## 2. Case Presentation

### 2.1. Initial Diagnosis

A 26-year-old male presented with a Class III malocclusion, having been repeatedly advised to undergo orthognathic surgery, which he categorically refused. Clinical examination revealed a bilateral full Class III molar relationship with anterior crossbite, reduced incisal overbite, particularly involving the lower left lateral incisor, which appeared in infraocclusion and marked extrusion of the upper right second molar, likely related to the full-step Class III malocclusion on the right and the absence of occlusal contact with the opposing lower right second molar. Transversely, mandibular endoalveolia suggested a compensatory adaptation to maxillary constriction. The lower incisors were retroclined, indicating dentoalveolar compensation of the crossbite. The facial profile was characteristic of a Class III pattern, with deficient malar projection and mandibular prominence, within a hyperdivergent skeletal context. Lingual posture was low and anterior at rest.

Cephalometric analysis confirmed a skeletal Class III relationship, with maxillary dentoalveolar protrusion and mandibular dentoalveolar retrusion. The patient exhibited a negative overjet and a minimal overbite. Given the patient’s refusal of surgical intervention, a dentoalveolar compensatory treatment plan was selected, aiming to correct the anterior crossbite and improve interarch relationships while maintaining vertical dimension control and addressing esthetic considerations.

**Figure 1:**
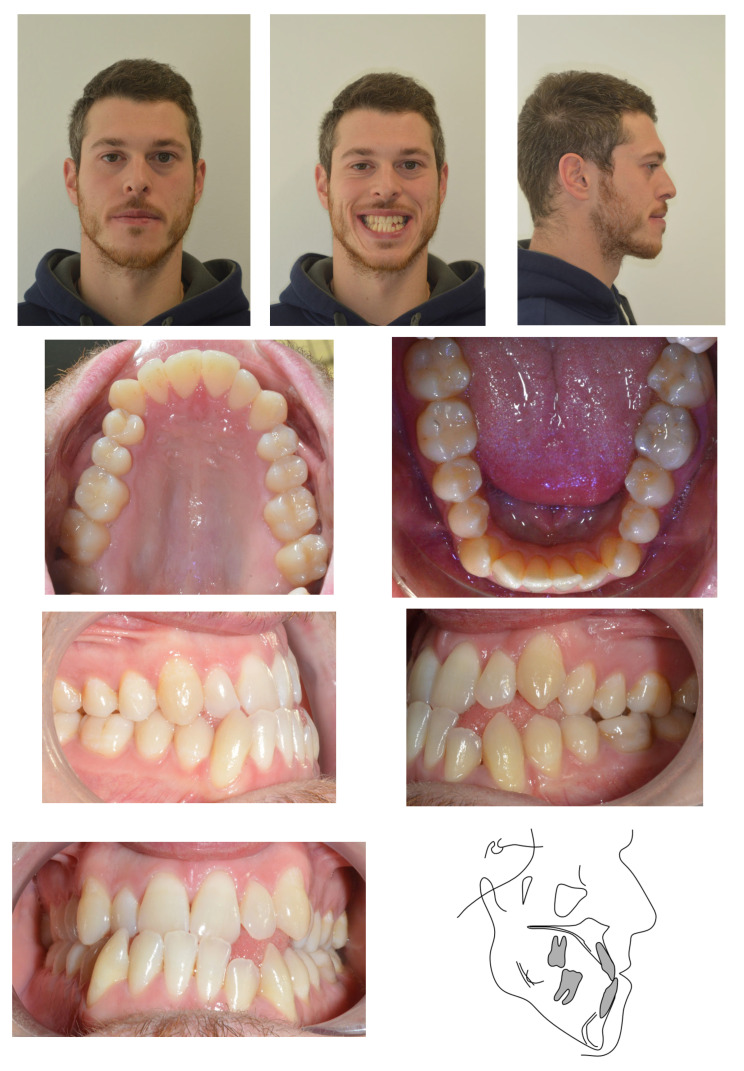

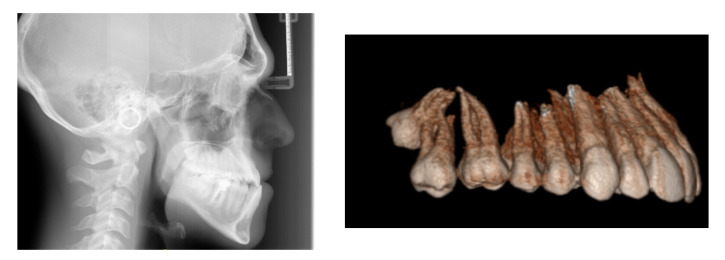
Initial records.

Medical history was unremarkable, with no systemic diseases, drug allergies, or ongoing medications. No relevant family history of Class III malocclusion or significant maxillofacial dysmorphology was reported (Figure 1).

### 2.2. Treatment Objectives

In accordance with the “Sagittal First” philosophy developed by Dr. Carriere, treatment began with the placement of a Carriere^®^ Motion Class III appliance, bonded from the lower right first premolar to the lower right second molar. The “Sagittal First” philosophy is a treatment sequencing strategy that prioritizes correction of the anteroposterior (AP) discrepancy at the very beginning of orthodontic therapy, before comprehensive alignment with brackets or aligners. This concept, as applied with the Carriere^®^ Motion 3D appliance, allows clinicians to address the most challenging sagittal component first and then proceed with definitive alignment and detailing, improving predictability and facilitating finishing with aligners or fixed appliances.

This device was combined with a series of Invisalign^®^ aligners in the maxillary arch, serving as active anchorage for intermaxillary elastics, with buttons bonded on the upper first molars. This setup provided simultaneous control of the maxillary arch and contributed to sagittal correction. The primary objective of this initial phase was to reestablish favorable intermaxillary relationships early in treatment, while aligning and coordinating the upper arch. In a subsequent phase, once a solid Class I molar occlusion was achieved, full-arch clear aligner therapy was planned to optimize alignment, close lower arch spaces, and ensure final arch coordination, especially in the vertical dimension.

### 2.3. Treatment Progress

**Figure 2:**
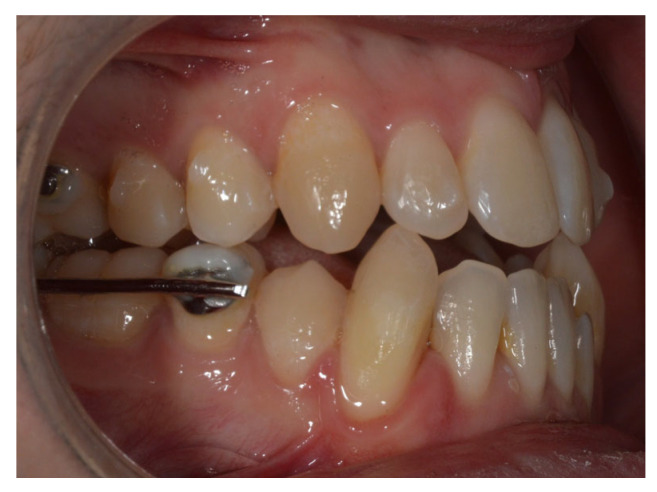
Intraoral view of the Carriere^®^ Motion 3D Class III appliance during the sagittal correction phase.

**Figure 3:**
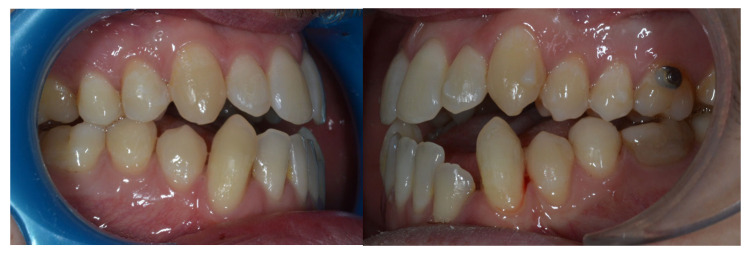
Evolution of treatment after the use of the Carriere appliance with Sagittal First philosophy.

Treatment began with the extraction of the upper right second molar, performed by the referring general dentist. Once the Carriere^®^ Motion appliances were bonded from the lower right first premolar to the lower right second molar and from the lower left first premolar to the lower left first molar, and the maxillary Invisalign^®^ aligners were in place, the first phase of Class III intermaxillary elastics was initiated using ¼″, 6 oz elastics for one month, then intensified with 3/16″, 8 oz elastics (Figure 2). Achievement of a bilateral Class I molar relationship marked the transition to the second phase of treatment (Figure 3).

**Figure 4:**
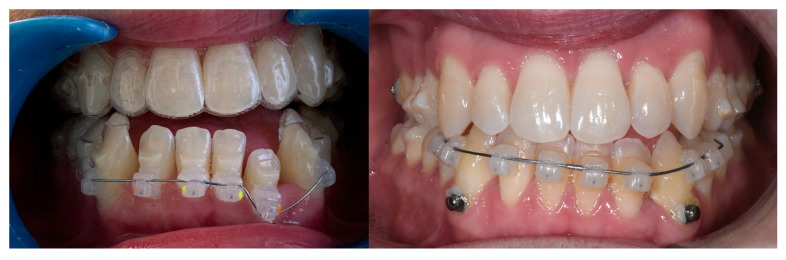
Changes in the vertical position of the mandibular incisor (32) achieved through hybrid treatment involving the use of brackets.

Due to persistent difficulties in extruding the lower left lateral incisor during the initial full set of lower aligners and the lack of response after a second attempt (refinement), a hybrid approach was adopted. A sectional fixed appliance was placed from the lower left canine to the lower right canine using conventional brackets to facilitate the extrusion of the lower left lateral incisor, a movement that had proven ineffective with aligners alone in this case (Figure 4). Concurrently, ¼″, 4 oz Class III elastics were maintained to reinforce sagittal corrections and minimize relapse risk.

Active treatment lasted 32 months and required six consecutive series of aligners. At the end of treatment, customized fixed retention was placed from canine to canine in both arches.

### 2.4. Treatment Results

**Figure 5:**
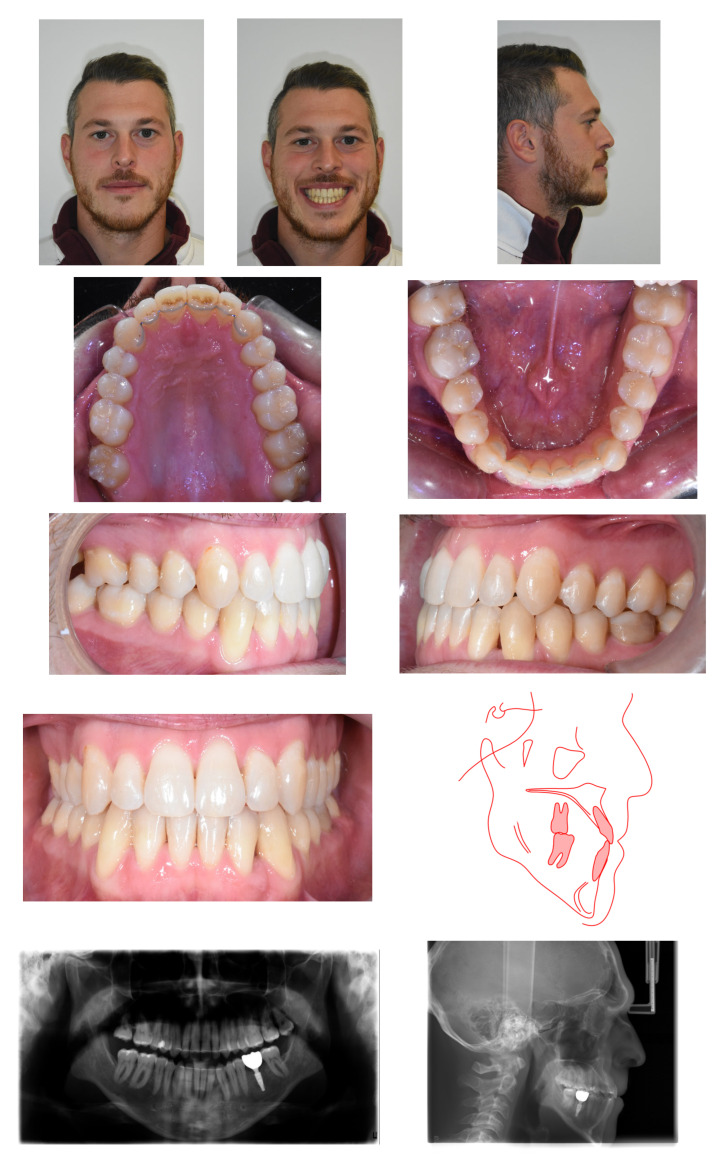
Final records.

A Class I dental relationship for both canines and molars was achieved, with centered maxillary and mandibular midlines. Periodontally, a loss of the interdental papilla between the lower left lateral incisor and the lower left canine was noted. Radiographs were obtained following the loss of the lower left first molar due to pulpitis, which was subsequently replaced with an osseointegrated implant and a ceramic crown (Figure 5).

Post-treatment cephalometric analysis revealed an improvement in the skeletal Class III relationship attributed to clockwise mandibular rotation, evidenced by increased vertical divergence (Table 1). This had a positive effect on the sagittal projection of the chin. This is consistent with the mechanism of vertical compensation of a sagittal problem, as seen in the MEAW technique and also with the Class III Carriere^®^ Motion Distalizer as an alternative approach.

**Figure 6:**
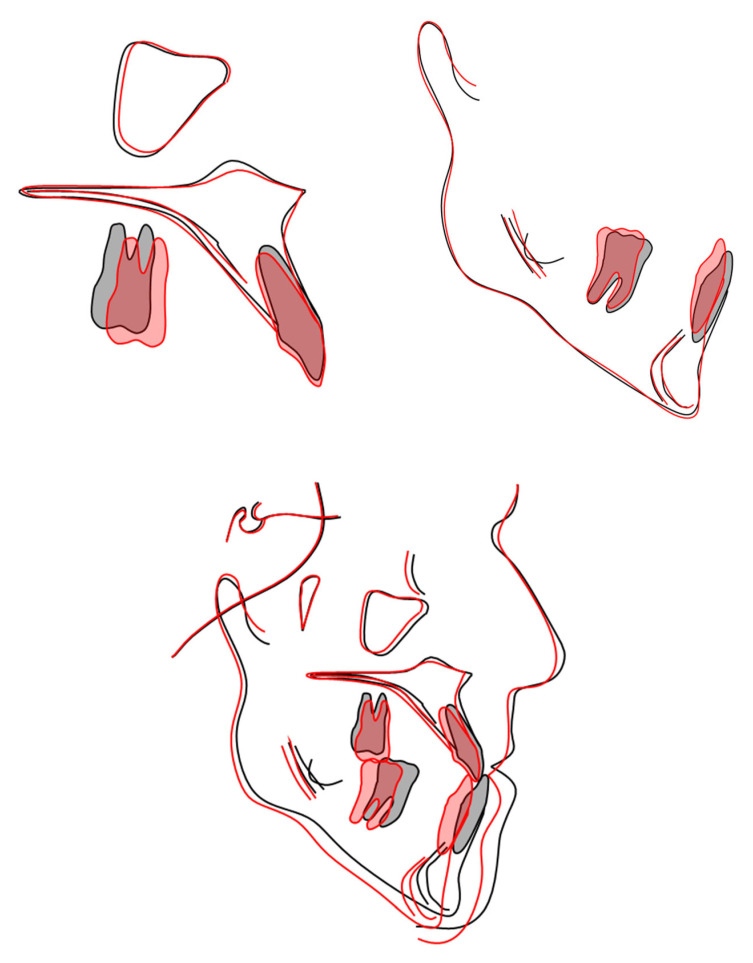
Patient structural superimposition using Björk’s method.

Dentoalveolar changes effectively compensated for the Class III malocclusion, with almost any maxillary incisor protrusion, and significant mandibular incisor extrusion and retroclination, improving interarch relationships. The upper molars showed extrusion in order to compensate for the change in the occlusal plane induced by the use of CLIII elastics. The lower molars showed distal inclination of the crown, consistent with the dental correction of the CLIII relationship. The overjet was corrected from −1.7 mm to 2.7 mm, and the overbite was maintained at 1.7 mm (previously 1.9 mm), ensuring stable occlusal harmony (Table 1, Figure 6).

The upper right third molar replaced the extracted upper right second molar, although a slight open bite remained.

For retention, two custom-bonded wires were chosen to allow the upper right third molar to gradually establish optimal contact with its antagonist.

The patient was very satisfied with the esthetic and functional result and particularly appreciated avoiding surgery (Table 2).

## 3. Discussion

Cephalometric analysis indicates that the improvement of the sagittal discrepancy was mainly attributable to dentoalveolar compensation, including mandibular incisor retroclination, lower molar distal tipping and maxillary incisor proclination, while skeletal changes were limited and consisted predominantly of a mild clockwise mandibular rotation, as reflected by the increase in SN-MP angle. The use of the Carriere^®^ Motion Class III appliance facilitated dentoalveolar compensation via anterior extrusion and distalization of the lower arch, which, given the inclination of the patient’s mandibular body, promoted this clockwise rotation. Such dentoalveolar effects of the Carriere^®^ Motion Class III appliance have already been demonstrated in the literature and are consistent with previous reports showing primarily dentoalveolar changes with limited skeletal modification in adult Class III patients [10]. Furthermore, the use of clear aligners specifically designed for tooth movement, incorporating attachments, provided superior anchorage compared to a simple Essix retainer and enabled the execution of orthodontic movements from the earliest stages in the upper arch. However, limitations were observed in the use of aligners for certain movements, such as extrusion of the lower left central incisor, which proved unpredictable [13,14]. This finding confirms a predictable limitation of aligner-only mechanics for specific movements and justifies the adoption of a hybrid approach combining clear aligners with a sectional fixed appliance to achieve the desired correction. Furthermore, the present report is based on a single compliant adult patient; therefore, the clinical outcomes described herein should be interpreted with caution and cannot be generalized to all orthodontic cases. The hybrid approach combining brackets and auxiliaries like the Carriere^®^ Motion appliance represented a suitable compromise between esthetics and treatment efficiency. Nonetheless, in such cases, it is essential to inform patients about the limitations of compensating a surgical Class III malocclusion with orthodontic camouflage alone.

As this report is limited to a single clinical case, it is important to clarify the indications and limitations of this non-surgical camouflage approach. Even though normo and hypodivergent skeletal Class III patterns appear to be more favorable candidates for orthodontic compensation, the approach may also work in hyperdivergent cases like the one presented in this paper. The use of Class III elastics guides extrusion of the anterior terminal of the Carriere appliance (typically the lower canines or first premolars), thereby inducing a counterclockwise rotation of the occlusal plane. In the phase following use of the Carriere Motion Class III appliance, the lower anterior teeth are extruded to conform to the newly established occlusal plane. This new occlusal plane may result in a slight clockwise rotation of the mandible, which can have a positive impact on the Class III facial profile by reducing chin prominence through a backward and downward repositioning of the mandible [10]. Patient motivation and expectations are of paramount importance: the orthodontist must clearly inform the patient about the inherent limitations of camouflage treatment, including the modest impact on facial profile and the potential risk of relapse when orthognathic surgery is avoided.

## 4. Conclusions

Severe skeletal Class III malocclusion in an adult refusing surgery was successfully camouflaged using the Carriere^®^ Motion 3D Class III appliance combined with clear aligners and a short hybrid phase. The Sagittal First approach rapidly established Class I molar relationships, and final clear aligner therapy with sectional fixed mechanics achieved stable occlusion, positive overjet/overbite, and functional improvement through clockwise mandibular rotation. Despite limited predictability of pure aligner extrusion, this minimally invasive protocol offers an effective and esthetic non-surgical alternative for motivated patients.

## Figures and Tables

**Table 1:** Changes in the patient’s cephalometric values.

Name	Measurement Unit	Normal Value	Std. Deviation	Initial	Final
Skeletal Analysis					
Angle SNA	°	82.0	2.0	85.4	84.6
Angle SNB	°	80.0	2.0	88.5	86.9
ANB	°	2.0	4.0	−3.1	−2.4
SN-MP-abo	°	32.0	6.0	32.6	34.6
FMA-abo	°	25.0	4.0	30.0	31.8
Dental Analysis					
+1i/NA	mm	4.0	1.0	8.7	9.6
+1/SN	°	103.0	4.0	112.2	113.8
−1i/NB	mm	4.0	1.0	6.4	3.8
−1/MP-abo	°	99.0	1.0	76.8	73.6
Overjet	mm	3.5	2.5	−1.7	2.7
Overbite	mm	2.0	2.5	1.9	1.7
Facial Analysis					
Ls/E-line	mm	−4.0	2.0	−6.9	−5.2
Li’/E-line	mm	−2.0	2.0	−2.1	−1.9

**Table 2:** Treatment timeline.

Time	Event
Month 0	- Initial records - Extraction of tooth 17 (upper right second molar) - Bonding of bilateral Carriere^®^ Motion 3D Class III appliances (34–36 and 44–47) - Delivery of first series of maxillary Invisalign^®^ aligners with bonded buttons on 16 and 26
Months 0–1	Class III elastics ¼″, 6 oz (full-time wear)
Month 1–6	Switch to Class III elastics 3/16″, 8 oz (full-time wear)
Months 12–18	- Achievement of bilateral Class I molar and canine relationships - Removal of Carriere^®^ Motion appliances - Start of full-arch Invisalign^®^ treatment (upper and lower) + first refinement series
Months 19–31	Successive refinement series (total of 6 aligner series completed)
Month 32	- End of active treatment - Removal of all appliances - Placement of fixed lingual retainers 13–23 and 33–43 - Delivery of removable retention trays - Final records
>Month 36	Loss of tooth 36 (pulpitis) → implant placement + ceramic crown

## Data Availability

The original data presented in the study are included in the article, further inquiries can be directed to the corresponding author.
